# Relationship between Sjogren's syndrome and gastroesophageal reflux: A bidirectional Mendelian randomization study

**DOI:** 10.1038/s41598-024-65512-4

**Published:** 2024-07-04

**Authors:** Jie Liu, Jiali Li, Guanghui Yuan, Tingting Cao, Xiaojin He

**Affiliations:** 1 Department of Rheumatology and Immunology, Jiangsu Province Hospital of Chinese Medicine, Affiliated Hospital of Nanjing University of Chinese Medicine, No.155, Hanzhong Road, Qinhuai District, Nanjing, Jiangsu Province China; 2 Department of Gastroenterology, Jiangsu Province Hospital of Chinese Medicine, Affiliated Hospital of Nanjing University of Chinese Medicine, No.155, Hanzhong Road, Qinhuai District, Nanjing, Jiangsu Province China

**Keywords:** Mendelian randomization, Gastroesophageal reflux, Sjogren's syndrome, Causal relationship, Immunology, Applied immunology, Immunogenetics

## Abstract

The clinical incidence of sjogren's syndrome combined with gastroesophageal reflux disease is high. Existing observational studies have shown inconsistent results in the association between gastroesophageal reflux disease (GERD) and Sjogren's syndrome (SS).We observed that the symptoms of SS patients also improved after receiving GERD-related treatment. Therefore, we aimed to investigate the relationship between GERD and SS through a bidirectional two-sample Mendelian randomization (MR) study. Independent SNPs associated with GERD and SS were selected from a genome-wide association study (GWAS) as instrumental variables to conduct a bidirectional two-sample Mendelian analysis of GERD and SS. Genetic data were obtained from two databases for the following two outcomes: Gastroesophageal reflux (IEU Open GWAS) [sample size = 602,604 (patients = 129,080; nonpatients = 473,524)] and SS (FinnGen) [sample size = 392,423 (patients = 2,495; nonpatients = 389,928)]. Statistical methods for the MR analysis included the inverse-variance weighting method, weighted median, simple mode and weighted mode, as well as heterogeneity and sensitivity analyses using the Cochran Q statistic, MR‒Egger regression, outlier detection methods (MR-PRESSO). In addition, Steiger Test was conducted to test the direction of causality. MR analysis showed a positive correlation between GERD and SS risk [odds ratio (OR) = 1.3279 (95% confidence interval 1.0312–1.7099, *P* = 0.0280)]. However, in contrast, no significant causal effect of SS on GERD was observed [OR = 1.0024 (95% CI 0.9651–1.0412; *P* = 0.8995)]. This bidirectional two-sample Mendelian randomization study confirmed a causal relationship between SS and GERD, and suggested that GERD is a risk factor for SS, while SS does not affect GERD.

## Introduction

Sjogren's syndrome (SS) is a chronic multisystem autoimmune disease characterized by decreased salivary and lacrimal gland function^1^. When the condition occurs independently without any accompanying diffuse connective tissue disease, it is referred to as primary Sjögren's syndrome (pSS)^2^. Epidemiological studies have shown that the overall global prevalence of primary Sjogren's syndrome is 60.82 patients/100,000 inhabitants, with a female/male ratio of 10.72^3^. The main characteristics of Sjogren's syndrome are sicca symptoms^4^, which can also occur respiratory, digestive, nervous and other system diseases^5^.Interestingly, we have found that patients with Sjogren's syndrome often present with symptoms such as reflux and heartburn during clinical treatment, and the results of previous studies are consistent with our observations.GERD is one of the common complications of SS. The rate of oesophageal motor abnormalities and gastroesophageal reflux symptoms in SS patients is 33–60%^6,7^.However, due to the influence of confounding factors, it is difficult to distinguish the causal relationship between the two diseases in clinical studies, and researchers have given different opinions. We observed that patients with Sjogren's syndrome did not improve their GERD related symptoms after receiving the treatment regimen for SS, and some patients experienced an aggravation of reflux due to drug side effects. Confusingly, when SS patients received medication to treat GERD, their condition improved at the same time. We speculate whether there is some genetic link between GERD and SS, and GERD as a risk factor affects the occurrence and development of SS.

Mendelian randomization (MR) is a widely used method. It is based on Mendel's second law^8^, utilizing genetic variants that are closely related to exposure to obtain more reliable evidence^9^. Mendelian randomization (MR) can help us further investigate the degree of association between exposure and the occurrence of a disease by mitigating confounding factors and related interferences that could affect causal relationships^10^. Therefore, we conducted a bidirectional two-sample MR study to explore the potential causal relationship between GERD and SS.

## Materials and methods

This study was registered in the OSF database (https://osf.io/), and the research protocol and analysis data can be obtained from the following website: 10.17605/OSF.IO/CU3W9. Additionally, our research was reported in accordance with the STROBE-MR reporting guidelines^11^. All data used in this study are publicly available summary statistics from genome-wide association studies (GWAS). Therefore, no additional ethical approval or informed consent was needed.

### MR study assumptions and design

**Figure 1:**
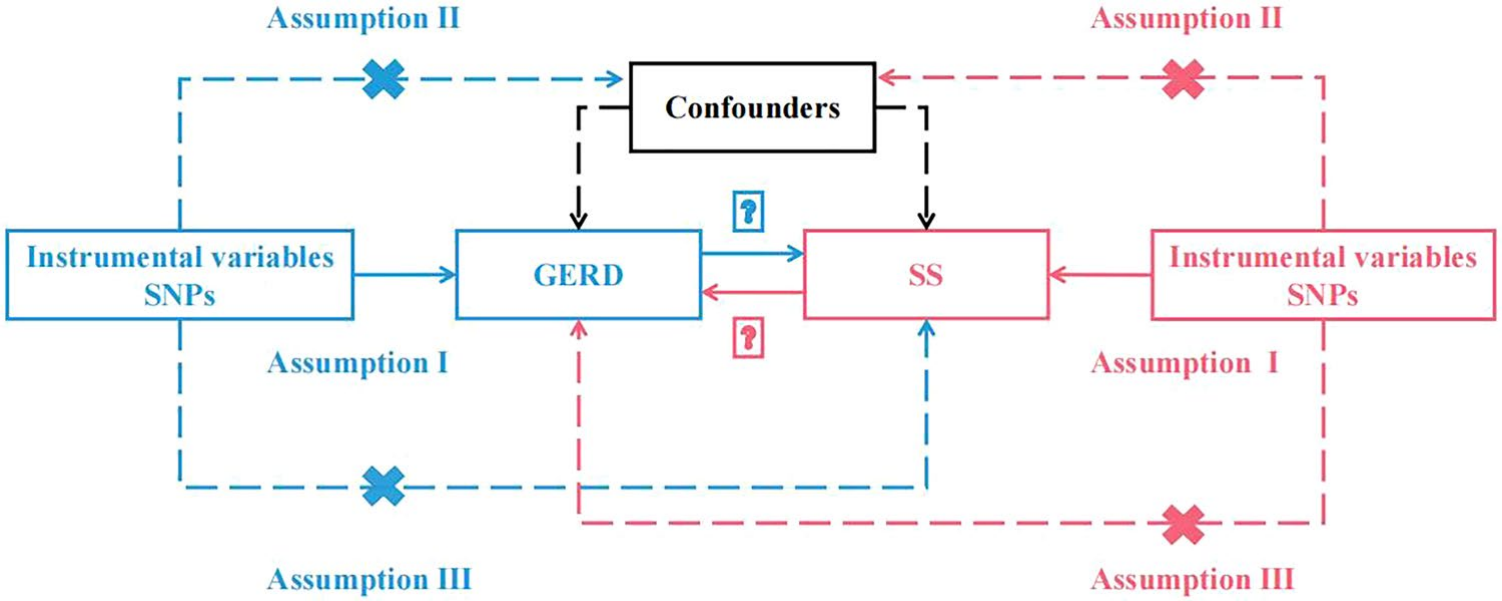
Diagram of critical assumptions for MR analysis.

A bidirectional two-sample Mendelian randomization (MR) study was performed to assess the causal relationship between GERD and SS. Figure 1 illustrates the flowchart of the MR study between GERD and SS. In this study, summary data from genome-wide association studies (GWAS) were utilized, where single nucleotide polymorphisms (SNPs) significantly associated with SS and GERD were used as instrumental variables to estimate the causal effects of the exposure variables. MR analysis relies on three core assumptions to ensure the validity of the results: (1) the relevance assumption, indicating a strong correlation between instrumental variables and exposure; (2) the independence assumption, implying the lack of association between instrumental variables and all confounding factors; and (3) the exclusion-restriction assumption, suggesting that instrumental variables do not have a direct association with the outcome but only influence it indirectly through the exposure. Instrumental variables play a pivotal role in MR studies, and thus sensitivity analyses, MR‒Egger regression, Cochran’s Q test, MR-PRESSO, and other methods were employed to detect and correct bias arising from genetic heterogeneity and horizontal pleiotropy.

### Data sources

The summary data for GERD were obtained from a recent large-scale GWAS, which included 602,604 participants mainly of European ancestry (GERD patients/control individuals: 129,080/473,524)^12^. These data can be found in the IEU Open GWAS project database^13^. Additionally, summary data for Sjögren's syndrome were acquired from the FinnGen biobank (https://www.finngen.fi/en),^14^ and the phenotype code "M13_SJOGREN" yielded a total of 392,423 samples (SS patients/control individuals: 2,495/389,928; SS patient sex distribution: 2,155 females/340 males), with 20,170,011 SNPs genotyped. SS patients were defined based on International Classification of Diseases (ICD) codes, including ICD-10 code M35.0, ICD-9 code 7102, or ICD-8 code 73,490 (mostly based on ICD-10 codes). Information for each data source is presented in Table 1.

**Table 1:** Details of the GWAS summary-level data.

Phenotype	Sample size		SNP(n)	Ancestry	Data accession address
	N case	N control			
GERD	129,080	473,524	2,320,781	European	https://gwas.mrcieu.ac.uk/
SS	2495	389,928	20,170,011	European	https://www.finngen.fi/en

SS: Sjogren’s syndrome; GERD: Gastroesophageal reflux disease; SNP: Single nucleotide polymorphisms.

### Selection and assessment of instrumental variables

Based on the three assumptions of MR mentioned above, we first referred to the whole genome information of the European 1000 Genome Project. Using R Studio 4.3.1 software, we filtered out SNPs strongly associated with GERD from the database (*P* < 5 × 10^–8^), which satisfied the relevance assumption. It should be noted that for the exposure related to SS, due to the limited number of SNPs (n = 1) obtained when using a strict *P* value threshold (*P* < 5 × 10^−8^), a more lenient threshold (*P* < 5 × 10^−6^) was recommended to extract instrumental variables. The F-statistic can measure the strength of the relationship between genetic variation and exposure. Therefore, we calculated the F-statistic to assess the presence of weak instrumental variable bias (F < 10) and further validated the relevance assumption. We calculated the F and R^2^ values of each SNP as follows: ${\text{F}} = \frac{{{\text{R}}^{2} \times \left( {{\text{n}} - 2} \right)}}{{1 - {\text{R}}^{2} }}$ (n: sample size of the GWAS; R^2^: the proportion of explained variance of the IV), ${\text{R}}^{2} = \frac{{2 \times\upbeta ^{2} \times (1 - {\text{EAF}}) \times {\text{EAF }}}}{{2 \times\upbeta ^{2} \times (1 - {\text{EAF}}) \times {\text{EAF}} + {\text{SE}}^{2} \times 2 \times N \times {\text{EAF}} \times (1 - {\text{EAF}})}}$ (β: estimate of the genetic effect of each SNP on iron status; EAF: effective allele frequency; SE: β standard error; N: sample size of the GWAS)^15–17^. Next, the linkage disequilibrium parameter^18^ (LD-r^2^) threshold was set to 0.001, and the clumping distance threshold was set to 10,000 kb to remove SNPs (LD-r^2^ > 0.001 with the most significant SNP) within 10,000 kb, which ensured the independence of the SNPs from each other. The online tool PhenoScanner V2^19^ was employed to exclude SNPs associated with other confounding factors, satisfying the independence assumption and the exclusion-restriction assumption. The exposure and outcome datasets were merged, containing instrumental variables' relationships with both the outcome and exposure. Then, the palindromic SNPs were removed. The SNPs obtained after the abovementioned steps of screening were considered the final instrumental variables representing the exposure.

### MR analysis

Multiple methods were employed in this study to determine the causal relationship between SS and GERD. Prior to analysis, exposure and outcome data were harmonized to align the effect alleles with the positive strand. If the number of SNPs was less than 3, the inverse variance-weighted method (IVW) under a fixed-effects model was used. For exposures with three or more SNPs, the IVW method under a multiplicative random-effects model was utilized as the primary statistical method. Additionally, MR‒Egger, weighted median, simple mode, and weighted mode were used for supplementary analyses. Cochran's Q statistic was employed to assess heterogeneity among the estimated values of each SNP. If *p* < 0.1 for Q, it suggests the presence of potential heterogeneity. Sensitivity analyses were performed, including weighted median, MR‒Egger regression and MR-pleiotropy residual sum and outlier methods (MR-PRESSO), to assess robustness and detect horizontal pleiotropy. If no horizontal pleiotropy is detected, IVW results are considered reliable^20^. Based on the assumption that the effect of genetic variation pleiotropy on the outcome is independent of the effect of genetic variation on exposure (InSIDE), in MR‒Egger regression, the intercept is used to test for genetic pleiotropy, while the slope provides the causal estimate between exposure and outcome. If the intercept included the null hypothesis (close to 0), the MR‒Egger results are consistent with those of IVW, which indicates that there is an absence of horizontal pleiotropy or that the effect of genetic variation pleiotropy is small^21^. The weighted median estimator can provide a reliable estimate if more than 50% of the instrumental variables are valid. Compared to IVW, the weighted median has less bias and a lower type-I error rate, thus serving as a supplement to MR‒Egger results^22^. In a set of parallel measurements, if certain data differ significantly from the mean value, then these data are regarded as doubtable values, also called outliers. The existence of outliers affects the reliability of the results, so we additionally used MR-PRESSO to detect the presence of abnormal value variation. If any outliers were detected, they were discarded, and MR analysis was repeated after correction to reduce causal estimate bias. Additionally, Steiger Test was conducted to avoid reverse causality^23^. The Steiger Test assessed whether SNPs were more associated with outcomes than exposure. SNPs with a “FALSE” direction might not be associated with exposure and should be removed before MR analysis.To prevent reliance on a single SNP or potential biases, a leave-one-out method was performed, calculating the effect value of the remaining instrumental variables after removing each SNP. The software packages "TwoSampleMR" and "MRPRESSO" from R version 4.3.1 were used for each analysis. Finally, the statistical power of the MR analysis was computed based on GWAS sample size, the proportion of patients, the effect size of the association between exposure and outcome (OR) and R^2^. The process was carried out online at https://shiny.cnsgenomics.com/mRnd/, with an alpha level of 0.05.

## Ethical approval

Data were collected from public databases. The FinnGen study was approved by the Coordinating Ethics Committee of the Helsinki and Uusima Hospital Districts. All procedures performed in studies involving human participants comply with the ethical standards of institutional and/or national research councils as well as the 1964 Declaration of Helsinki and its later amendments or similar ethical standards.For GERD GWAS, this research was approved by the QIMR Berghofer's Human Research Ethics Committee under project ID 3501.

## Results

### The causal effect of GERD on SS

Genetic instrumental variables strongly associated with GERD were retrieved from the database, and information on GERD instrumental variables in outcome (SS) was extracted. Then, the exposure and outcome data were integrated to generate a dataset for MR analysis. Finally, a total of 65 independent SNPs were identified as genetic instrumental variables for GERD, and detailed information is listed in Supplementary Table 1. Palindromic variants causing potential allelic ambiguity were excluded, as shown in Supplementary Table 2. The F-statistics for IVs were all above 10, indicating that IVs were generally considered to provide sufficient information for the MR study. In the meta-analysis of estimates from IVW, the summary OR for SS of genetically predicted per log-OR increase in GERD was 1.42 (95% CI 1.04–1.92, *p* = 0.0248). Meanwhile, the results of MR Egger, weighted medium, simple mode, and weighted mode were consistent with the results of IVW, although the four methods mentioned above were not statistically significant (*p* < 0.05). The ORs for each method (MR‒Egger, weighted median, simple mode, and weighted mode) were 0.5115 (95% CI 0.0800–3.2668, *p* = 0.4811), 1.2224 (95% CI 0.8497–1.7586, *p* = 0.2791), 1.0757 (95% CI 0.4421–2.6172, *p* = 0.8727), and 1.1926 (95% CI 0.6109–2.3282, *p* = 0.6074), respectively, as shown in Table 2.

**Table 2:** Causal effects of GERD on SS in MR analysis.

Exposure	Outcome	nSNP	Method	Beta	*P*-value	OR	95%CL
GERD	Sjogren syndrome	65	Inverse variance weighted	0.3478	0.0248	1.4159	(1.0449, 1.9186)
GERD	Sjogren syndrome	65	MR Egger	-0.6704	0.4811	0.5115	(0.0801, 3.2669)
GERD	Sjogren syndrome	65	Weighted median	0.2008	0.2791	1.2224	(0.8497, 1.7585)
GERD	Sjogren syndrome	65	Simple mode	0.0730	0.8727	1.0757	(0.4421, 2.6172)
GERD	Sjogren syndrome	65	Weighted mode	0.1762	0.6074	1.1927	(0.6109, 2.3282)

SS: Sjogren’s syndrome; GERD: Gastroesophageal reflux disease; SNP: Single nucleotide polymorphisms; CI: Confdence intervals.

**Figure 2:**
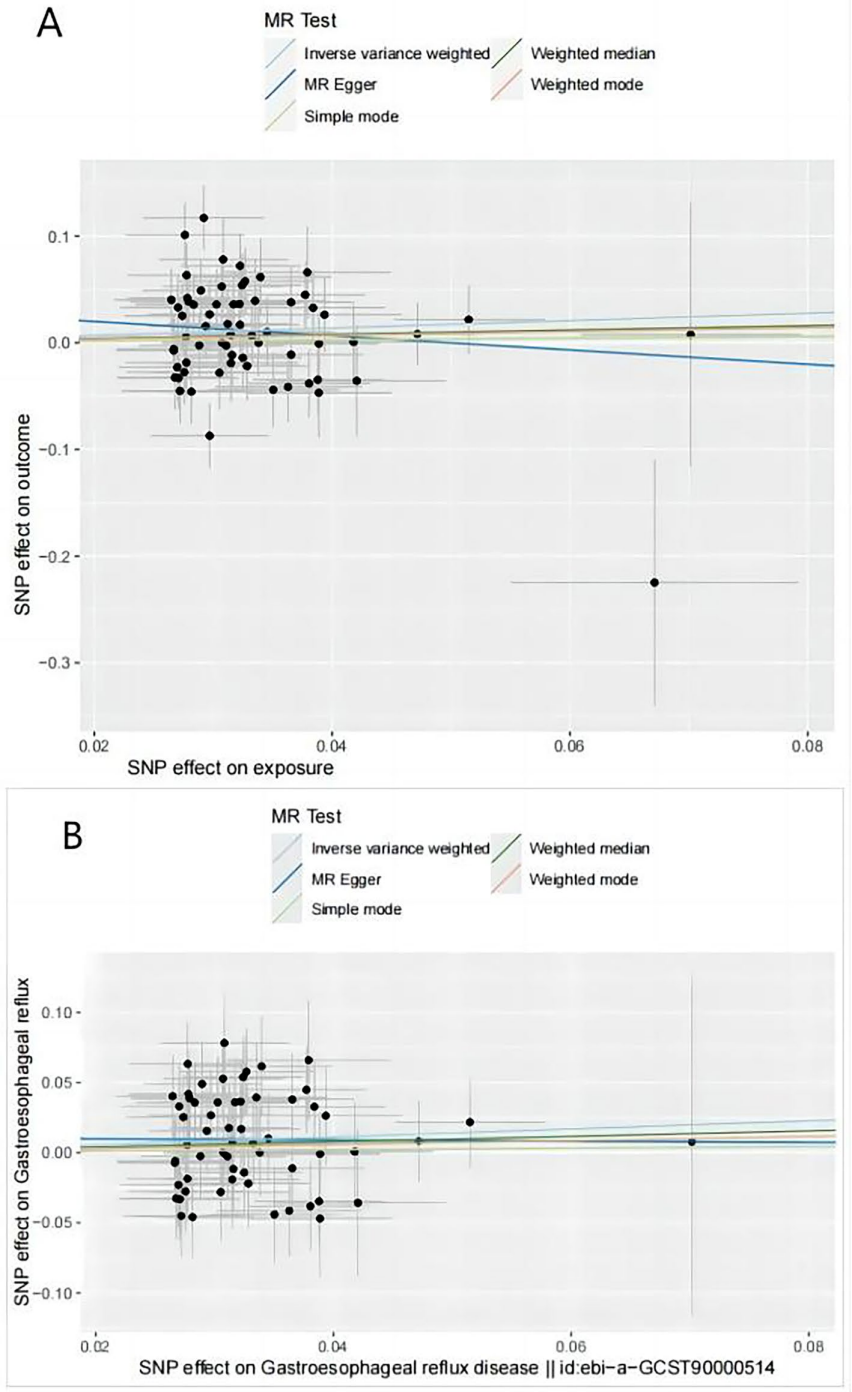
Scatter plot of the causal effect of GERD on SS.(**A**).Initial MR Analysis results. (**B**).MR Analysis results corrected for outliers.

In addition, we conducted several sensitivity analyses and heterogeneity analyses to assess pleiotropy and potential genetic outliers. The heterogeneity of each SNP estimate was evaluated by Cochran's Q test, with Q statistics of 104.25 and 106.22, calculated by MR‒Egger and inverse-variance weighting, respectively. The p value for Q was not statistically significant (*p* < 0.1), indicating the presence of genetic heterogeneity. This can be visualized in the funnel plot, as shown in *Supplementary Fig. 1*. Based on this, we further analysed the data using MR PRESSO, and the Global Test showed a *p* value of 0.0014 (< 0.05), indicating the presence of heterogeneity. Moreover, a potential outlier for GERD in the IEU Open GWAS database was identified: the 36th SNP, rs2744961. Therefore, potential pleiotropy was assessed through the Global Test. However, the distortion test showed that this outlier did not influence the direction of the results, with a *p* value of 0.6878 (≥ 0.05). Additionally,based on the leave-one-out sensitivity plot and forest plot, SNPs which had significant impact on the results were screened and regarded as possible outliers, such as rs12453010, rs4713692, rs6722661 and rs3828917. Finally,Steiger Test indicated that each SNP used for GERD explained more variance in GERD than in SS. After outlier correction, we found that the MR results remained similar. As listed in Table 3, the meta-analysis result of the IVW estimate showed an OR of 1.3279(95% CI 1.0312–1.7099, *p* = 0.0280) for GERD and SS. The OR values obtained from the other four statistical methods were 0.9594(95% CI 0.2009–4.5823, *p* = 0.9587), 1.2155(95% CI 0.8445–1.7496, *p* = 0.2936), 1.0547(95% CI 0.4175–2.6647, *p* = 0.9107) and 1.1563(95% CI 0.5473–2.4428, *p* = 0.7049). The intercept term test in MR‒Egger did not suggest pleiotropic effects (*p* = 0.6812 > 0.05). Cochrane’s Q value was recalculated.The result showed there was no heterogeneity between the data (Q = 63.3717,63.5581, *p* = 0.2927,0.3191). The individual SNP effects and combined effects of each MR method were visualized in scatter plots [Fig. 2]. Leave-one-out plots and forest plots indicated that these associations were unlikely to be driven by extreme SNPs [*Supplementary Fig. 1*]. Finally, after filling in the relevant data on the online website [sample size of GWAS (602,604), proportion of patients (0.21) and effect size OR (1.33), R^2^(0.0040), alpha level set to 0.05], we calculated the statistical power of this MR analysis to be 1.00. Based on the above analysis results, we infer that gene-predicted GERD is positively associated with the risk of SS.

**Table 3:** Causal effects of GERD on SS in MR analysis(after correcting for outliers).

Exposure	Outcome	nSNP	Method	Beta	*P*-value	OR	95%CL
GERD	Sjogren syndrome	60	Inverse variance weighted	0.2836	0.0280	1.3279	(1.0312, 1.7099)
GERD	Sjogren syndrome	60	MR Egger	-0.0415	0.9587	0.9594	(0.1060, 3.5682)
GERD	Sjogren syndrome	60	Weighted median	0.1952	0.2936	1.2155	(0.8647, 1.7173)
GERD	Sjogren syndrome	60	Simple mode	0.0533	0.9107	1.0547	(0.4665, 2.4609)
GERD	Sjogren syndrome	60	Weighted mode	0.1452	0.7049	1.1563	(0.5577, 2.5054)

SS: Sjogren’s syndrome, GERD: Gastroesophageal reflux disease, SNP: Single nucleotide polymorphisms, CI: Confdence intervals.

### The causal effect of SS on GERD

**Figure 3:**
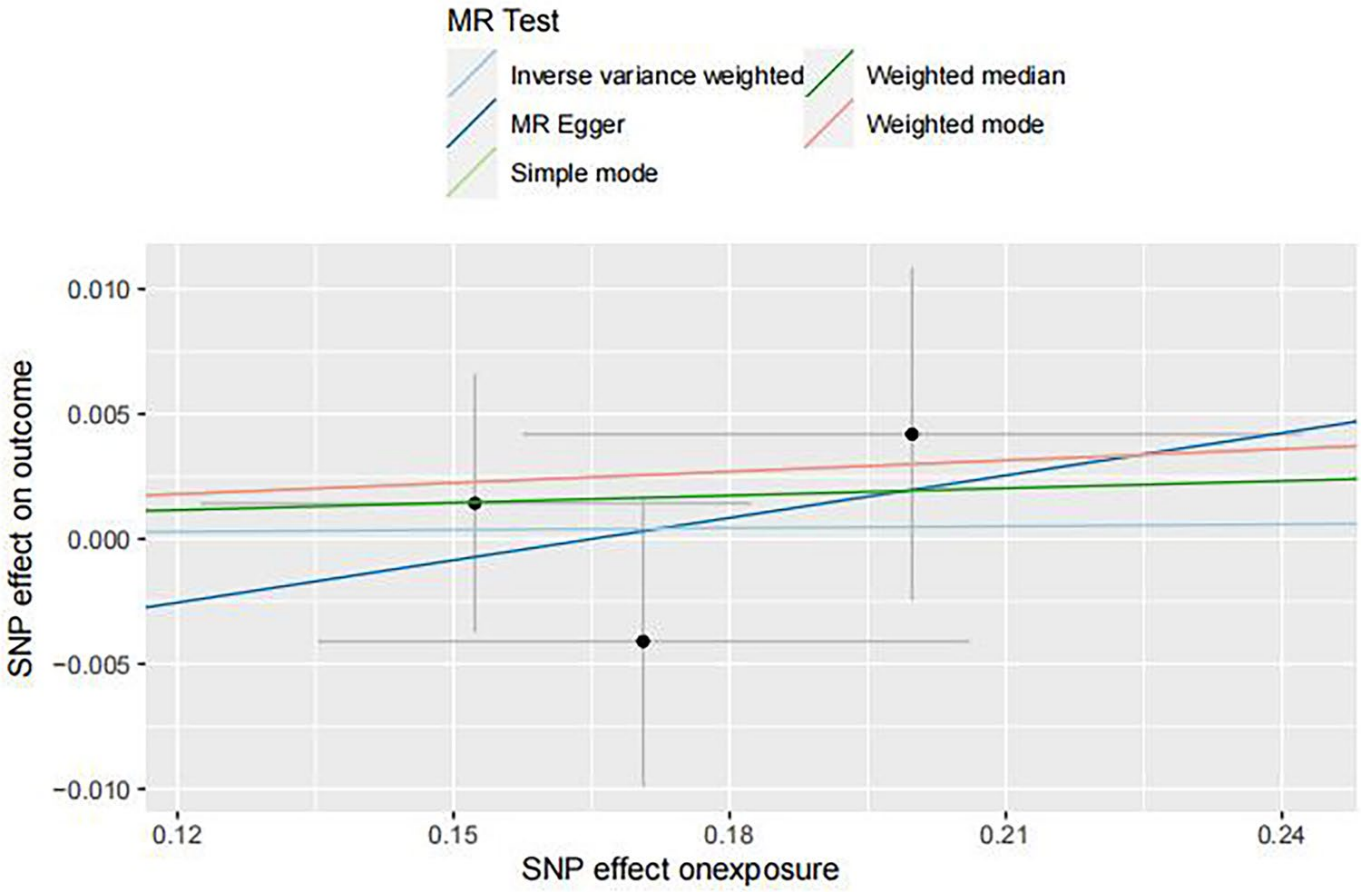
Scatter plot of the causal effect of SS on GERD.

SS was used as an exposure factor to demonstrate reverse causality, with reverse MR having a lower statistical power of 0.05, and the results are presented in Table 4 and Fig. 3. To include more SNPs related to SS, this study used a more lenient threshold (*P* < 5 × 10 − 6). One palindromic SNP, rs4145584, which could potentially cause allelic ambiguity, was excluded. Finally, three SNPs were obtained for the MR analysis of the causal relationship between SS and GERD, and detailed information can be found in Supplementary Table 3. Five methods were also used for statistical analysis (IVW, MR‒Egger, weighted median, simple mode, and weighted mode). The ORs of SS associated with GERD were 1.0024 (95% CI 0.9651–1.0412; *p* = 0.8995), 1.0581 (95% CI 0.7492–1.4945; *p* = 0.8022), 1.0097 (95% CI 0.9639–1.0577; *p* = 0.6828), 1.0151 (95% CI 0.9586–1.0749; *p* = 0.6589), and 1.0151 (95% CI 0.9592–1.0742; *p* = 0.6556), respectively. Similarly, heterogeneity analysis was conducted, and the results showed no heterogeneity among the data, as the Cochran's Q test was not statistically significant (Q = 0.9680202, *p* = 0.6163070). Additionally, there was no evident directional pleiotropy in either the MR‒Egger or MR-PRESSO Global Test (MR‒Egger intercept: *p* = 0.809 > 0.05; MR-PRESSO Global Test: *p* = 0.792 > 0.05). The results mentioned above indicated that gene-predicted SS was not significantly associated with the risk of GERD (all *p* > 0.05). The Steiger Test was performed for all SNPs and each SNP included was with a “TRUE” direction in the test.Furthermore, the leave-one-out analysis plot and forest plot demonstrated that the effects of each SNP on the outcome were generally consistent, indicating the stability of our results (Supplementary Fig. 2).

**Table 4:** Causal effects of SS on GERD in MR analysis.

Exposure	Outcome	nSNP	Method	Beta	*P*-value	OR	95%CL
Sjogren syndrome	GERD	3	Inverse variance weighted	0.0024	0.8995	1.0024	(0.9651, 1.0412)
Sjogren syndrome	GERD	3	MR Egger	0.0565	0.8023	1.0582	(0.7492, 1.4945)
Sjogren syndrome	GERD	3	Weighted median	0.0097	0.6828	1.0097	(0.9639, 1.0577)
Sjogren syndrome	GERD	3	Simple mode	0.0150	0.6589	1.0151	(0.9586, 1.0749)
Sjogren syndrome	GERD	3	Weighted mode	0.0150	0.6556	1.0151	(0.9592, 1.0743)

GERD: Gastroesophageal reflux disease, SNP: Single nucleotide polymorphisms, CI: Confdence intervals.

## Discussion

As a systemic autoimmune disease, SS disease can affect the digestive system during its progression, leading to conditions such as gastroesophageal reflux disease, chronic gastritis, pancreatitis, and autoimmune hepatitis^24^. However, it is difficult to establish a causal relationship between SS and these conditions, as some patients may already have developed them at the time of pSS diagnosis. Recent prospective cohort studies have shown that patients with SS have a 2.41-fold increased risk of developing GERD in the Chinese population^25^. However, Another report by Grande et al. indicated that there was no correlation between oesophageal motility patterns and SS, and no consistent pattern existed^26^. We have observed that SS patients’ symptoms can be alleviated after receiving treatment related to GERD. However, the symptoms of GERD in SS patients do not improve when they only receive treatment for the primary disease, and some patients have aggravated regurgitation due to drug side effects. Based on these findings, we speculated that gastroesophageal reflux may be a risk factor affecting the occurrence and development of SS. Surprisingly, the results of this Mendelian randomization analysis also confirmed this speculation: there may be a causal relationship between GERD and SS, and GERD is considered to be a risk factor for SS from the genetic perspective, while the reverse is not true.

One possible explanation for the conflicting conclusions could be that some observational studies are unable to avoid the influence of confounding factors on the accuracy of the association results, such as shared immune response pathways, among others. Additionally, due to the diversity and nonspecificity of clinical symptoms for both SS and GERD, many patients may remain undiagnosed or misclassified, which could contribute to result biases. However, despite these limitations, our MR analysis, along with previous studies, reached a consensus that there is an association between SS and GERD.

The exact mechanisms underlying the association between SS and GERD are not fully understood. Existing studies have given several explanations for the concomitance of GERD in SS patients. From the perspective that SS precedes GERD: As we are well aware, the pathophysiology of GERD is multifactorial. Various mechanisms may contribute to the symptoms of GERD, including gastric anatomy and motility, anti-reflux barrier, characteristics of refluxate, clearance mechanisms, mucosal integrity, and symptom perception^27^. Previous observational studies have suggested that SS may induce GERD symptoms based on the above mechanisms. Several studies have shown that pSS patients exhibit a significantly reduced average trunk length percentage of LES and resting LES pressure^28^. As a result, when intra-abdominal pressure rises above the pressure in the LES region, patients are more prone to experience reflux. When reflux occurs, the oesophagus undergoes dilation and secondary peristalsis through neural reflexes to achieve clearance under normal physiological conditions. However, in pathological conditions, reduced oesophageal motility strength impairs the ability to empty refluxed material. Reports have indicated that 35% of pSS patients exhibit abnormal oesophageal motility patterns, including ineffective oesophageal motility, "nutcracker" oesophageal motility, and nonspecific oesophageal motility disorders. Second, primary Sjögren's syndrome affects exocrine glands, primarily salivary glands, leading to a significant reduction in saliva production, which can cause difficulty in swallowing food^29^. The acid exposure time of the oesophagus is significantly prolonged, increasing the incidence of oesophagitis to some extent. Another mechanism that cannot be ignored is the immune-inflammatory response, Park et al.^30^ found that antibodies in pSS patients can inhibit cholinergic neurotransmission mediated by muscarinic acetylcholine receptors, leading to decreased oesophageal motility and altered colonic motility, inhibition of gastrointestinal smooth muscle movement, delayed gastric emptying resulting in proximal gastric distension, and induction of transient relaxation of the lower oesophageal sphincter. Then, a vicious cycle of acid exposure-decreased oesophageal clearing ability is formed. Additionally, chronic immune inflammation in the glands may significantly reduce the inherent ability of the mucosa of the pharynx, larynx, and oesophagus to protect themselves from acid and gastric protease damage. Tissue resistance depends on protective mucin from the cellular layer, which limits the diffusion of hydrochloric acid and buffers the pH. When the glands and related tissues undergo an immune storm and inflammation, the mucosa of the pharynx, larynx, and oesophagus may not be able to respond well to the damaging effects of gastric reflux^31^. Furthermore, in terms of medication, exogenous factors such as nonsteroidal anti-inflammatory drugs (NSAIDs) can exacerbate stimulation and damage to the patient's oesophagus. In clinical trials, common medications for pSS, such as glucocorticoids, NSAIDs, and immunosuppressants, have been reported to carry risks of varying degrees of gastrointestinal adverse reactions. Finally, the development of GERD may be partially related to psychological factors. Studies have shown that anti-NMDA-NR2A/B antibodies in pSS patients can activate glutamate receptors^32^, leading to central sensitization, and stimuli below the normal threshold can cause reactions and discomfort. pSS patients often experience varying degrees of swallowing difficulties and delayed gastric emptying. Based on existing research findings, we recognize the distinct pathophysiology of the gastroesophageal tract in SS patients, which naturally leads to the assumption of a causal impact of SS on GERD. However, cross-sectional and cohort studies often do not exclude confounding factors, which may result in selection bias. Furthermore, there is no definitive conclusion regarding the sequence of occurrence between SS and GERD, and thus the causal relationship between the two diseases remains elusive. This study investigates the issue from a genetic standpoint, and the results indicate that SS does not have a relevant impact on GERD, which is markedly different from previous reports.

From the perspective that GERD precedes SS: In recent years, the role of gastroesophageal reflux in inducing autoimmune diseases has garnered widespread attention. Two longitudinal follow-up studies using national sample cohorts^33^ have indicated a bidirectional association between GERD and rheumatoid arthritis (RA), with an increased risk of RA in GERD patients across all age and gender subgroups. A subsequent Mendelian randomization study^34^ suggested that GERD could induce the onset of RA in the European population, while RA had no effect on GERD. A retrospective analysis^35^ showed that patients with Sjögren's Syndrome (SS) involving digestive impairment appeared to have a more severe phenotype. Is there a causal relationship between GERD and SS as well? Similar to the findings mentioned above, our study indicates an association between GERD and SS, and having GERD may increase the incidence of SS. GERD could potentially increase the risk of SS by altering the microbiota of the gut and esophagus. The study results by Qian Jun et al.^36^ showed that in patients with GERD, there was a significant increase in the relative abundance of Bacteroides and Prevotella, and a significant decrease in Actinobacteria, Micrococci, Lactobacilli, Streptococci, and Rotella. The changes in the microbiota of SS patients were consistent with these findings^37^. GERDSZYMULA and others^38^ hypothesized that molecular mimicry of bacterial peptides from oral commensals (digesting Prevotella lytoltica, codigenet sputum, codigenet flavus) in SS patients could induce an immune response by activating SSA/Ro60 reactive T cells, which may be a potential trigger for autoimmunity in SS patients. VAN DER MEULEN et al.^39^ speculated that the oral microbiota might activate TLRs through PAMPs, leading to the occurrence of autoimmunity, and the presence of PAMPs in tissues may be associated with autoimmunity. Therefore, the dysbiosis of the gastrointestinal microbiota following gastroesophageal reflux disease could increase the risk of Sjögren's Syndrome by modulating autoimmunity and inflammation.

Research on the association between GERD and the risk of developing SS is currently lacking. To our knowledge, this is the first bidirectional MR study evaluating the causal relationship between GERD and SS. In this study, several steps of MR analysis were conducted to satisfy three core assumptions. Regarding assumptions one and two, the instrumental variables were closely related to the exposure and unrelated to any potential confounding factors. We set both threshold and genetic distance threshold limitations when extracting instrumental variables from the GWAS database, ultimately selecting 60 and 3 SNPs closely associated with GERD and SS, respectively, with each SNP being independent of each other. To validate assumption three and ensure the robustness and evidence strength of the MR analysis results, sensitivity analysis and heterogeneity analysis were used to detect and correct potential pleiotropy. It was reassuring that no bias from sources of pleiotropy was found.

This study is designed based on clinical observation, and its results will also have a reference for clinical diagnosis and treatment: 1. We should be alert to the possibility of SS when we find GERD patients. 2. Doctors can first improve the symptoms of GERD in SS patients to control the progression of SS. In the future, we can further study the efficacy and mechanism of GERD therapeutics to improve SS. However, there are still some potential issues and limitations that cannot be resolved in this MR analysis. First, even though we relaxed the screening threshold for SS-related SNPs, the number of SNPs selected was still limited. Although there were no weak instrumental variables (F statistics were all greater than 10), this may have resulted in low statistical power. Second, these GWAS data did not include specific individual information, so we did not perform further subgroup analysis. The population in this study is of European descent, which avoids false associations introduced by population stratification, but it also limits the extrapolation of the results to other ethnicities or races. The global GWAS database is constantly evolving, and in the future, it will be necessary to supplement the sample size and sample information to replicate this study in more racially/ethnically diverse populations to validate the causal association between the two diseases.

## Conclusions

In conclusion, this MR study confirmed the genetic association between SS and GERD and suggested that GERD is a risk factor for SS, while SS does not affect GERD. Existing studies have elucidated some pathological mechanisms underlying the association between SS and GERD, but further research is needed to clarify the pathophysiology of the causal relationship between the two.

### Supplementary Information

Supplementary Information.

## Data Availability

The genetic data of Gastroesophageal reflux and SS are available at IEU Open GWAS (https://gwas.mrcieu.ac.uk/datasets/ebi-a-GCST90000514/) and FinnGen (https://storage.googleapis.com/finngen-public-data-r9/summary_stats/finngen_R9_M13_SJOGREN.gz). Supplementary files or related sources are described in the manuscript.

## Abbreviations

SS: Sjogren’s syndrome
MR: Mendelian randomization
GWAS: Genome-wide association studies
IVW: Inverse-variance weighted
GERD: Gastroesophageal reflux disease
IV: Instrumental variable
SNP: Single nucleotide polymorphism
RCT: Randomized controlled trial
LD: Linkage disequilibrium
EAF: Efect allele frequency
CI: Confdence interval
MR-PRESSO: MR-pleiotropy residual sum and outlier methods
OR: Odds ratio
