# Supplementary material for: Relationship between Sjogren's syndrome and gastroesophageal reflux: A bidirectional Mendelian randomization study
Source: Sci Rep. 2024 Jul 4;14:15400. doi: 10.1038/s41598-024-65512-4 (PMC11224283; doi:10.1038/s41598-024-65512-4)

Supplementary Material

Supplementary Table 1. Summary of genetic variants (n=65) used to estimate the effect of gastroesophageal reflux disease on SS in MR analyses.

SNP	Chr	Pos (GRCh37)	Effect allele	Other allele	EAF	Beta	Se	P	R^2	F
rs1937450	1	66478840	G	T	0.537739	0.0315845	0.00484484	7.07E-11	7.05223E-05	42.4999
rs2782641	1	44013355	A	G	0.612669	0.0270882	0.00494583	4.33E-08	4.9777E-05	29.9972
rs2815749	1	72814783	G	A	0.800974	0.0388767	0.0060216	1.07E-10	6.91661E-05	41.6825
rs3766823	1	32197257	A	G	0.171467	0.0393599	0.00638545	7.09E-10	6.30472E-05	37.9947
rs569356	1	29136686	G	A	0.140835	-0.037919	0.00690971	4.07E-08	4.99735E-05	30.1157
rs7527682	1	189172684	G	A	0.53725	-0.026684	0.00482174	3.13E-08	5.08207E-05	30.6262
rs7541875	1	190957589	G	A	0.426069	0.0273972	0.00484976	1.61E-08	5.29562E-05	31.9132
rs903678	1	201809918	A	G	0.339412	0.0277378	0.00508463	4.89E-08	4.93823E-05	29.7594
rs1011407	2	60665768	G	A	0.121628	-0.0420618	0.00735917	1.09E-08	5.42079E-05	32.6676
rs12997558	2	41704580	A	G	0.358751	0.0278184	0.00502214	3.04E-08	5.09135E-05	30.6821
rs13409451	2	144257639	G	A	0.392403	-0.0277081	0.00493183	1.93E-08	5.23772E-05	31.5643
rs1596747	2	193802478	G	A	0.494136	0.0310869	0.00480742	1.00E-10	6.93854E-05	41.8147
rs4300861	2	22549441	T	C	0.38208	0.0307132	0.00494886	5.43E-10	6.39117E-05	38.5158
rs6711584	2	104421692	A	G	0.452019	0.0322545	0.00483974	2.66E-11	7.37008E-05	44.4155
rs6722661	2	100806588	A	G	0.364669	-0.0322543	0.00500354	1.15E-10	6.89538E-05	41.5546

rs7600261	2	212622818	T	C	0.306391	0.0338034	0.00522051	9.47E-11	6.95717E-05	41.9269
rs2240326	3	50128386	A	G	0.473775	-0.0471681	0.00481335	1.13E-22	0.000159331	96.0286
rs7612999	3	35678337	A	G	0.245338	0.0305231	0.00559546	4.90E-08	4.93778E-05	29.7566
rs10010963	4	159839313	T	C	0.616433	-0.0269803	0.00494665	4.92E-08	4.93649E-05	29.7489
rs13107325	4	103188709	T	C	0.074445	0.0701443	0.00918312	2.20E-14	9.68123E-05	58.3449
rs1510719	4	140938116	C	T	0.383439	-0.0388844	0.00494701	3.84E-15	0.000102515	61.7823
rs2164300	4	67813017	T	C	0.523279	-0.0264751	0.00482677	4.13E-08	4.99239E-05	30.0858
rs7675588	4	80734978	A	C	0.794635	-0.0335228	0.00595438	1.80E-08	5.2596E-05	31.6961
rs7685686	4	3207142	G	A	0.422353	-0.0279222	0.00489191	1.14E-08	5.40614E-05	32.5793
rs11953061	5	120144025	T	C	0.338908	0.0281599	0.00508694	3.10E-08	5.08504E-05	30.6441
rs329122	5	133864599	A	G	0.419631	-0.0289529	0.00488358	3.05E-09	5.83244E-05	35.1484
rs12204714	6	152235339	T	C	0.632223	-0.028817	0.00499422	7.92E-09	5.52466E-05	33.2936
rs2744961	6	34655000	T	C	0.358437	0.0292007	0.00501549	5.81E-09	5.62475E-05	33.8968
rs3828917	6	31465917	T	G	0.041826	0.0671113	0.0120054	2.27E-08	5.18541E-05	31.2490
rs4713692	6	33807638	T	C	0.367808	-0.0276128	0.00498634	3.07E-08	5.08866E-05	30.6659
rs9372625	6	98344031	A	G	0.383042	-0.037727	0.0049537	2.62E-14	9.62434E-05	58.0021
rs9373363	6	143150043	G	A	0.253631	-0.0326836	0.00555966	4.13E-09	5.73464E-05	34.5590
rs9396740	6	17023108	A	G	0.248794	-0.031493	0.0055587	1.47E-08	5.3263E-05	32.0981

rs11762636	7	2061111	A	C	0.180282	-0.0514827	0.00625598	1.88E-16	0.00011237	67.7221
rs2043539	7	12253880	A	G	0.41866	0.0272058	0.00486485	2.24E-08	5.18955E-05	31.2740
rs215614	7	32347335	A	G	0.629725	-0.0328541	0.00497715	4.08E-11	7.23027E-05	43.5729
rs2396133	7	109197067	G	A	0.475329	0.0293547	0.00481787	1.11E-09	6.16008E-05	37.1230
rs2396766	7	114318071	A	G	0.47308	0.0322057	0.00481878	2.34E-11	7.41185E-05	44.6673
rs3863241	8	73890335	T	C	0.52696	0.0324982	0.00481521	1.49E-11	7.55828E-05	45.5498
rs3793577	9	23737627	G	A	0.538279	0.0270309	0.00484976	2.49E-08	5.15498E-05	31.0656
rs4382592	9	134870755	G	T	0.699524	-0.0302679	0.00525091	8.20E-09	5.51366E-05	33.2272
rs7032155	9	122672771	A	C	0.59185	0.02775	0.00491358	1.63E-08	5.29267E-05	31.8954
rs1021363	10	106610839	G	A	0.641992	-0.031217	0.00502204	5.10E-10	6.41153E-05	38.6385
rs12357321	10	21790476	A	G	0.311087	0.0317159	0.00523077	1.33E-09	6.10049E-05	36.7639
rs761777	10	134938075	G	A	0.254034	0.0345341	0.00554469	4.71E-10	6.43698E-05	38.7919
rs2734839	11	113286490	T	C	0.606693	-0.0283478	0.00492778	8.79E-09	5.49137E-05	33.0929
rs7942368	11	76465362	T	C	0.214659	-0.0339683	0.00591917	9.54E-09	5.46475E-05	32.9325
rs1479405	12	15387519	T	C	0.3217	0.0314843	0.0051514	9.85E-10	6.19838E-05	37.3539
rs1716171	12	123716376	T	C	0.790024	0.0383981	0.00590382	7.83E-11	7.01926E-05	42.3012
rs324769	12	83969240	T	C	0.449179	-0.0267699	0.00483328	3.05E-08	5.09044E-05	30.6767
rs773109	12	56374695	A	G	0.335269	-0.0380572	0.00510213	8.71E-14	9.23205E-05	55.6377

rs1334297	13	58335375	A	G	0.734249	-0.0387984	0.00545512	1.14E-12	8.39365E-05	50.5845
rs9529055	13	66957533	A	G	0.475633	0.0266604	0.00481637	3.11E-08	5.08439E-05	30.6402
rs10133111	14	103377321	A	G	0.162996	0.0417875	0.00650789	1.35E-10	6.84149E-05	41.2298
rs942065	14	94032065	A	G	0.634045	0.0307384	0.00500925	8.45E-10	6.24824E-05	37.6544
rs9940128	16	53800754	A	G	0.421755	0.0332513	0.00486305	8.06E-12	7.75772E-05	46.7518
rs12453010	17	50316131	T	C	0.394803	0.0296967	0.00493315	1.75E-09	6.01325E-05	36.2382
rs12967855	18	35138245	G	A	0.670435	-0.0365451	0.00513354	1.09E-12	8.40922E-05	50.6784
rs1431196	18	50832102	G	A	0.428432	0.0324197	0.00486448	2.65E-11	7.37024E-05	44.4165
rs7241572	18	77580712	A	G	0.209101	0.0365511	0.00597464	9.49E-10	6.21039E-05	37.4262
rs2023878	19	18834124	T	C	0.192377	-0.0362846	0.00611913	3.04E-09	5.83456E-05	35.1612
rs9636202	19	18449238	A	G	0.26663	-0.035044	0.00547191	1.51E-10	6.80593E-05	41.0155
rs1883842	20	41223062	G	T	0.279255	0.0308332	0.00536826	9.27E-09	5.47412E-05	32.9889
rs2834005	21	34291708	C	T	0.315	0.0296997	0.0051734	9.42E-09	5.46885E-05	32.9572
rs9615905	22	48875699	T	C	0.458193	0.0275657	0.0048378	1.21E-08	5.3875E-05	32.4669

Note: SNP, single nucleotide polymorphism; Chr, chromosome; EAF, effect allele frequency; SE, standard error.

Supplementary Table 2. Summary of palindromic genetic variation of effects of gastroesophageal reflux disease on SS not included in MR Analysis (n=13).

SNP	Chr	Pos (GRCh37)	Effect allele	Other allele	EAF	Beta	Se	P	R2	F
rs17379561	1	98340139	T	A	0.144391	0.0530714	0.00686573	1.08E-14	9.91455E-05	59.7512
rs2358016	2	162007430	G	C	0.498113	0.028271	0.00481025	4.17E-09	5.73179E-05	34.5418
rs2016933	3	65653157	G	C	0.730053	-0.0310253	0.00542057	1.04E-08	5.43609E-05	32.7598
rs6780459	3	104624105	T	A	0.746622	0.0305507	0.0055211	3.14E-08	5.08086E-05	30.6189
rs1592757	5	103889998	C	G	0.355772	0.0311051	0.00502465	6.00E-10	6.35904E-05	38.3222
rs903959	8	142630782	A	T	0.399262	0.0291631	0.00491608	2.99E-09	5.83945E-05	35.1907
rs10837002	11	38565727	G	C	0.35122	0.0276491	0.00503665	4.03E-08	5.00062E-05	30.1354
rs9517313	13	99105892	C	G	0.383217	0.0331144	0.00494054	2.05E-11	7.45453E-05	44.9245
rs9542729	13	31833578	G	C	0.20244	-0.0363194	0.00599914	1.41E-09	6.08192E-05	36.6520
rs957345	14	75276079	G	C	0.540235	0.0290528	0.00482393	1.72E-09	6.01889E-05	36.2721
rs12598916	16	60658751	G	C	0.274798	-0.0332614	0.00539171	6.87E-10	6.31493E-05	38.0563
rs7206608	16	82872628	G	C	0.322927	0.0291541	0.005145	1.46E-08	5.32811E-05	32.1090
rs2838771	21	46501576	C	G	0.646721	-0.0280984	0.00506571	2.91E-08	5.10538E-05	30.7667

Note: SNP, single nucleotide polymorphism; Chr, chromosome; EAF, effect allele frequency; SE, standard error.

Supplementary Table 3. Summary of genetic variants (n = 3) used to estimate the effect of SS on gastroesophageal reflux disease in MR analyses.

SNP	Chr	Pos (GRCh37)	Effect allele	Other allele	EAF	Beta	Se	P	R^2	F	mr_keep
rs11250633	10	1568644	T	C	0.659	0.152319	0.0298015	0.00000032	6.65654E-05	26.1234	TRUE
rs17681310	6	6499325	A	G	0.1451	-0.199811	0.0422062	0.0000022	5.71093E-05	22.4122	TRUE
rs4145584	5	135222586	A	T	NA	0.218919	0.0470402	0.00000326	NA	NA	FALSE
rs7548516	1	151007805	C	T	0.2724	0.170587	0.0352744	0.00000132	5.95926E-05	23.3868	TRUE

Note: SNP, single nucleotide polymorphism; Chr, chromosome; EAF, effect allele frequency; SE, standard error.Palindromic SNP：rs4145584.

Supplementary Figure 1.Visualization of the results of MR Analysis of the effect of GERD on SS: funnel plot(A), forest plot(B), leave one method sensitivity analysis plot(C).

A

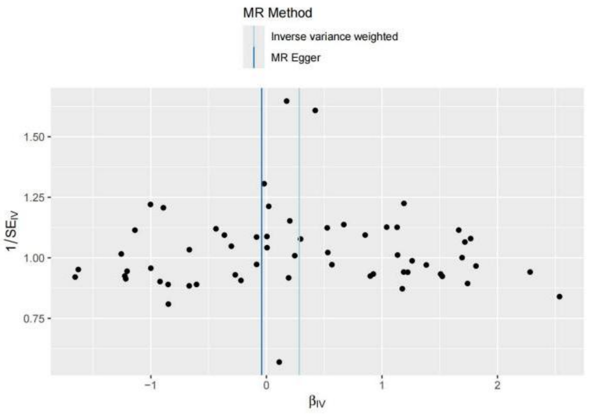

B

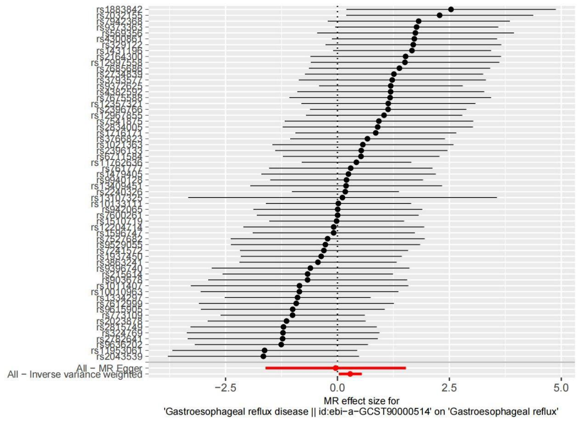

C

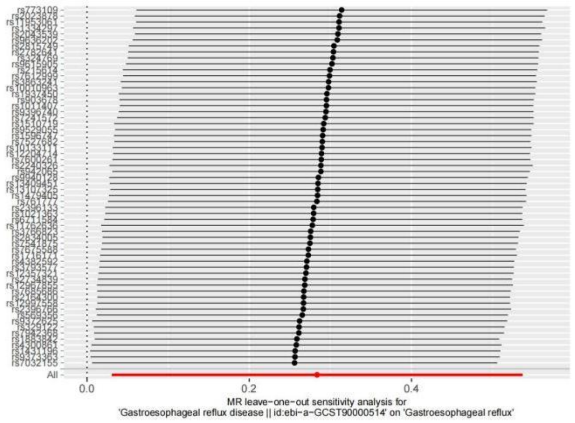

Supplementary Figure 2.Visualization of the results of MR Analysis of the effect of SS onGERD: forest plot(A), leave one method sensitivity analysis plot(B).

A

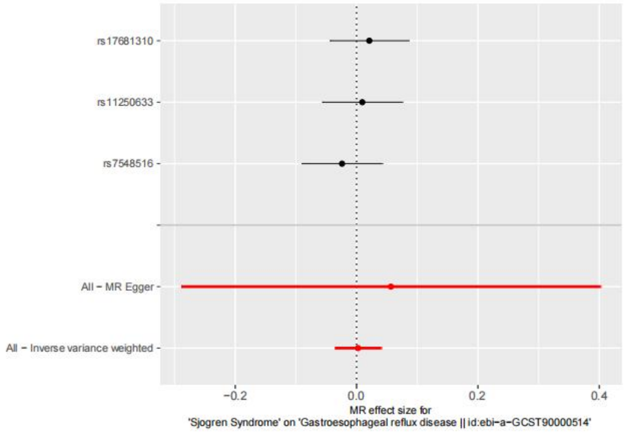

B

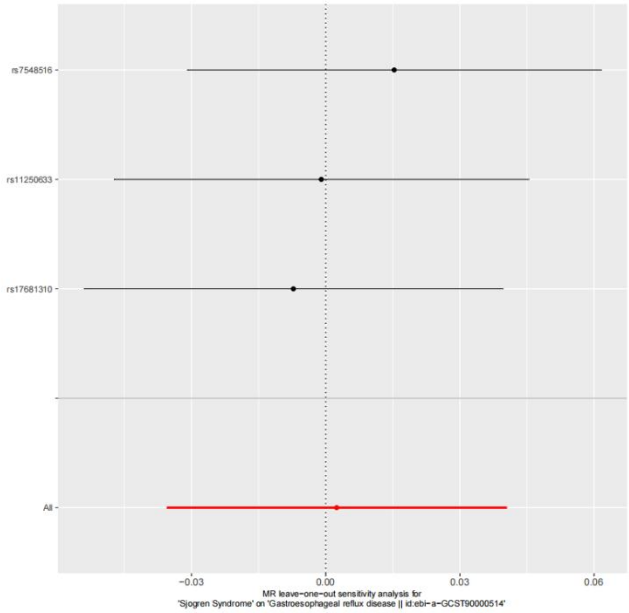

Supplement: Supplementary file 1 — Supplementary Information. [file 41598_2024_65512_MOESM1_ESM.pdf]
